# A pilot study of high-intensity interval training in older adults with treatment naïve chronic lymphocytic leukemia

**DOI:** 10.1038/s41598-021-02352-6

**Published:** 2021-11-30

**Authors:** Grace MacDonald, Andrea Sitlinger, Michael A. Deal, Erik D. Hanson, Stephanie Ferraro, Carl F. Pieper, J. Brice Weinberg, Danielle M. Brander, David B. Bartlett

**Affiliations:** 1 Division of Medical Oncology, Duke University School of Medicine, Durham, NC USA; 2 Duke Molecular Physiology Institute, Duke University School of Medicine, Durham, NC USA; 3 Hematologic Malignancies and Cellular Therapies, Duke University School of Medicine, Durham, NC USA; 4 Department of Exercise and Sport Science, University of North Carolina, Chapel Hill, NC USA; 5 Duke University Aging Center, Duke University School of Medicine, Durham, NC USA; 6 Division of Hematology, Duke University School of Medicine and VA Medical Center, Durham, NC USA; 7 Faculty of Health and Medical Sciences, University of Surrey, Guildford, UK; 8 Division of Medical Oncology, Department of Medicine, Duke Molecular Physiology Institute, Durham, NC 27701 USA

**Keywords:** Immunology, Physiology, Ageing, Haematological cancer

## Abstract

Chronic lymphocytic leukemia (CLL) is the most common leukemia in the USA, affecting predominantly older adults. CLL is characterized by low physical fitness, reduced immunity, and increased risk of secondary malignancies and infections. One approach to improving CLL patients’ physical fitness and immune functions may be participation in a structured exercise program. The aims of this pilot study were to examine physical and immunological changes, and feasibility of a 12-week high-intensity interval training (HIIT) combined with muscle endurance-based resistance training on older adults with treatment naïve CLL. We enrolled eighteen participants with CLL aged 64.9 ± 9.1 years and assigned them to groups depending on distance lived from our fitness center. Ten participants (4 M/6F) completed HIIT and six participants (4 M/2F) completed a non-exercising control group (Controls). HIIT consisted of three 30-min treadmill sessions/week plus two concurrent 30-min strength training sessions/week. Physical and immunological outcomes included aerobic capacity, muscle strength and endurance, and natural killer (NK) cell recognition and killing of tumor cells. We confirmed feasibility if > 70% of HIIT participants completed > 75% of prescribed sessions and prescribed minutes, and if > 80% of high-intensity intervals were at a heart rate corresponding to at least 80% of peak aerobic capacity (VO_2peak_). Results are presented as Hedge’s G effect sizes (*g*), with 0.2, 0.5 and 0.8 representing small, medium and large effects, respectively. Following HIIT, leg strength (*g* = 2.52), chest strength (*g* = 1.15) and seated row strength (*g* = 3.07) were 35.4%, 56.1% and 39.5% higher than Controls, respectively, while aerobic capacity was 3.8% lower (*g* = 0.49) than Controls. Similarly, following HIIT, in vitro NK-cell cytolytic activity against the K562 cell line (*g* = 1.43), OSU-CLL cell line (*g* = 0.95), and autologous B-cells (*g* = 1.30) were 20.3%, 3.0% and 14.6% higher than Controls, respectively. Feasibility was achieved, with HIIT completing 5.0 ± 0.2 sessions/week and 99 ± 3.6% of the prescribed minutes/week at heart rates corresponding to 89 ± 2.8% of VO_2peak_. We demonstrate that 12-weeks of supervised HIIT combined with muscle endurance-based resistance training is feasible, and that high adherence and compliance are associated with large effects on muscle strength and immune function in older adults with treatment naïve CLL.

Trial registration: NCT04950452.

## Introduction

With a median age at diagnosis of approximately 70 years, chronic lymphocytic leukemia (CLL) is the most prevalent adult leukemia in the USA^1–3^. The clinical presentation of CLL is diverse, and patients have a shorter life expectancy than age-matched healthy populations^4^. At present, there is no survival benefit from immediate or early therapy prior to established treatment indications, and most patients have a period of observation before therapy initiation^5,6^. During the treatment naïve period, normal immune functions are impaired, increasing the risk of secondary malignancies and infections^7–9^. Furthermore, treatment naïve patients have low physical fitness that predicts poor survival following commencement of treatment^10^.

One approach to concurrently improving physical fitness and immune functions may be participation in a structured exercise program^11,12^. Increasing physical activity levels and physical fitness are associated with improvements in therapy-related side effects, physical functioning, and quality of life for lymphoma patients^12–14^. Twelve weeks of moderate-intensity aerobic training improved physical function, fatigue, cardiovascular fitness, and QOL^14^, while 36-weeks of exercise training was effective at improving balance and quality of life^13^. However, these studies included 11.5% CLL patients^14^ and 46% B-cell Non-Hodgkin’s Lymphoma patients^13^. No study that we are aware of has assessed the effects of exercise training on important immune functions for CLL patients.

In healthy older adults, higher physical activity and physical fitness is mostly associated with better immune functions^11,15^. However, exercise-training interventions have more varied results, mainly because of the type, duration of and adherence to exercise. In clinical older adults, high-intensity interval training (HIIT) rapidly increases physical fitness, making it an attractive intervention for adults with low exercise capacity^16^. We, and others, have shown that HIIT in older patients with prediabetes or rheumatoid arthritis also reduces disease burden while improving important immune functions^16–20^. Therefore, HIIT may be an effective form of exercise to improve physical fitness and immune functions for CLL patients. Thus far, no study has assessed the effects of HIIT on physical fitness and relationships with immune functions in untreated CLL patients.

The aims of this pilot study were to examine physical fitness and immunological changes, and feasibility of a 12-week supervised HIIT intervention combined with muscle endurance-based resistance training in older adults with treatment naïve CLL. We used a HIIT intervention similar to one previously shown to provide immunomodulatory and aerobic fitness improvements^17,18,21^ and a resistance program designed to improve muscular strength^22^.

## Methods

**Figure 1:**
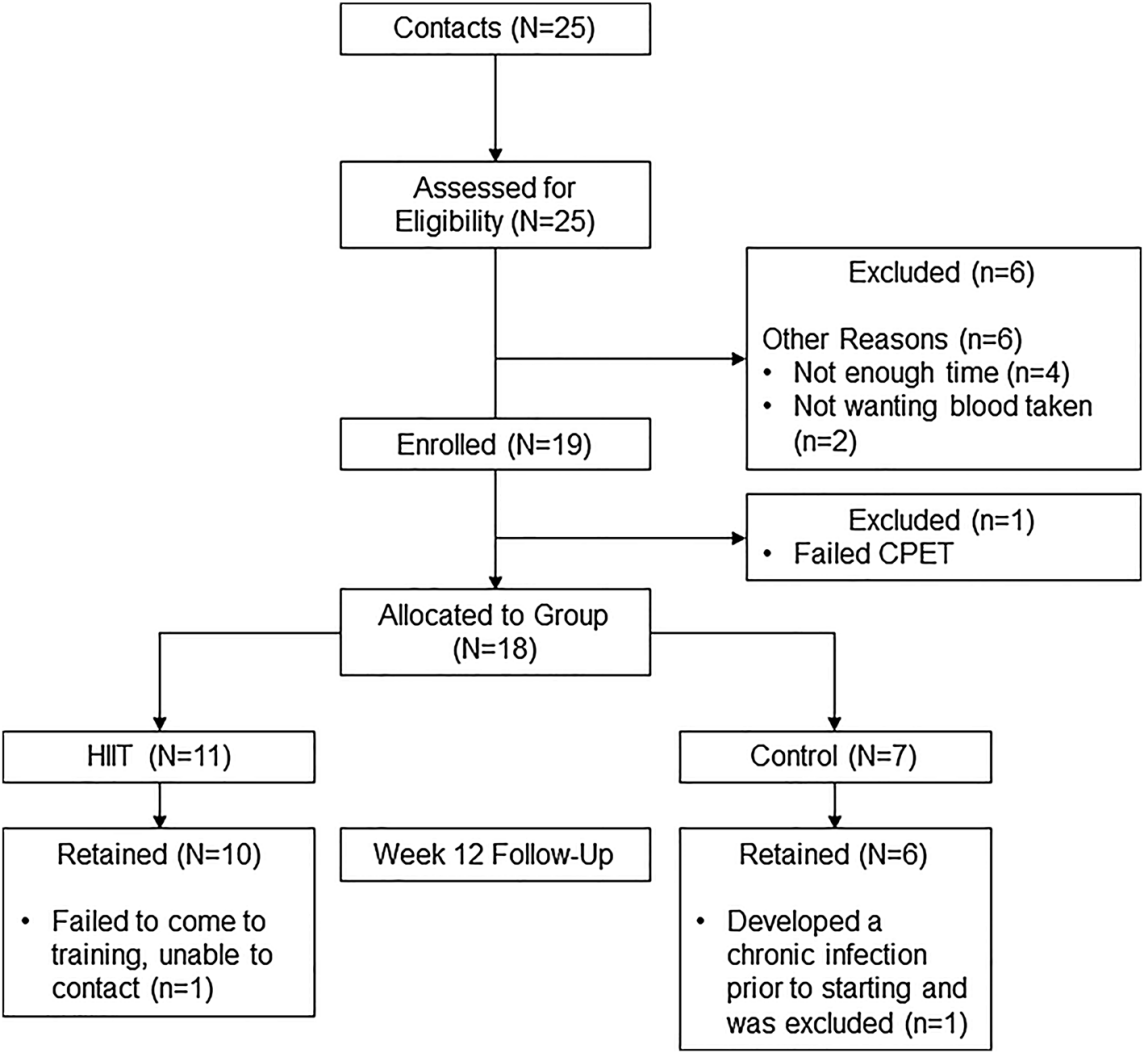
Consolidated standards of reporting trials (consort) diagram.

This study was conducted as a two-arm, quasi-experimental pilot study at Duke University between August 2018 and February 2020. Figure 1 diagrams the study participant progression. All participants provided written informed consent prior to study testing, and the study was approved by the Duke University Medical Center Institutional Review Board in accordance with the Declaration of Helsinki. Participants were non-randomized and allocated to the exercise group (HIIT) or the control group (Controls), depending on the distance they lived from our facility used for the supervised exercise training. If participants lived > 35 miles away, they were assigned to the control arm as this was deemed too far to travel three times per week.

### Eligibility

Eligibility requirements included (1) no abnormal cardiac findings observed during a maximal cardiopulmonary exercise test (CPET) that would prevent participants from exercising safely at high exercise intensities (see below for more details); (2) male or female with confirmed diagnosis of CLL as per the International Workshop on CLL Guidelines^23^; (3) no history of prior treatment of CLL; (4) able to walk on a treadmill; (5) no clinical evidence of significant disease progression with consideration for first line therapy within 6 months; (6) no systemic glucocorticoid therapy within the past 7 days; (7) no malignancy diagnosed within 3 years of study enrollment except for adequately treated basal, squamous cell carcinoma or non-melanomatous skin cancer, carcinoma in situ of the cervix, superficial bladder cancer not treated with intravesical chemotherapy or BCG within 6 months, localized prostate cancer; (8) no absolute contra-indications to exercise, including recent (< 6 months) unstable angina, uncontrolled dysrhythmias causing symptoms or hemodynamic compromise, symptomatic aortic stenosis, uncontrolled symptomatic heart failure, acute pulmonary embolus, acute myocarditis or pericarditis, suspected or known dissecting aortic aneurism, or acute systemic infection; (9) no orthopedic limitations, musculoskeletal disease and/or injury. Due to the nature of the study, participants with known joint, muscle or other orthopedic limitations that restrict physical activity were excluded; (10) no diabetes mellitus or chronic obstructive pulmonary disease; and (11) no uncontrolled hypertension (blood pressure ≥ 160/90).

### Fitness, strength, and body composition

*Cardiorespiratory fitness* We used a medically supervised cardiopulmonary exercise test (CPET) to assess cardiorespiratory fitness and cardiac health, including continuous 12-lead electrocardiogram assessment and breath-by-breath metabolic analyses (ParvoMedics, UT, USA) as previously described^17^. Aerobic capacity (VO_2peak_) was determined by a graded maximal treadmill test starting at 2 mph/0% grade with increasing speed and/or grade such that the metabolic demand increased at approximately 3.5 mL/kg/min until volitional exhaustion. We confirmed a valid test by either a respiratory exchange ratio (RER) of > 1.1 [mean RER: 1.2 (0.1)] or a rating of perceived exertion of ≥ 17 [RPE: mean 18 (1)]. We recorded blood pressure before, after the CPET, and at the end of each stage during the CPET. *Body composition* Total body mass, fat mass and lean mass were estimated by air-displacement plethysmography (BodPod System; Life Measurement Corporation, Concord, CA)^17^. *Muscle strength* We determined estimated maximal muscle strength and muscular endurance using three machine-weights—leg press, chest press, and seated row. At the initial visit, each exercise was explained to the participant and demonstrated by the exercise physiologist. Briefly, a warm-up set of 8–10 repetitions at 40–50% of predicted 1RM^24^, before a priming set of 5–7 reps at 50–60% 1RM was performed. Participants were then instructed to perform the maximum number of repetitions at 80–85% 1RM. If after the first attempt over 8 repetitions were performed, the weight was increased for the next set until no more than 8 repetitions could be completed. Participants were given 3–5 min rest in between each set. Predicted 1 RM was calculated and the participant performed as many repetitions as possible (muscular endurance) at 70% of 1 RM on each machine^25^. *Physical activity levels* We assessed participant’s habitual physical activity exposure by 7-day continuous wear of a wrist-based accelerometer (Garmin Vivosmart 3, Garmin, Kansas, USA) prior to and following the intervention. We assessed total steps/day during waking hours each day as a measure of habitual physical activity. Sleep time was determined by the device and confirmed by a sleep diary provided by each participant. All of the above tests and the blood samples were completed before training and 24 h after the participants last exercise session.

### 12-week exercise training program

The control group were informed to continue their daily activities and not participate in structured exercise programs. Participants assigned to the exercise group completed supervised training 3 times per week for 12-weeks. Two of the sessions were approximately 1 h, with 30 min for HIIT and 30 min for resistance training, and 1 session approximately 30 min for HIIT only (Table 1). Exercise intensities were determined from the CPET using VO_2_ reserve calculated as previously described^26^. Participants were given between 3 and 6 sessions to become accustomed to the exercise (30- to 45-s intervals at target heart rates, total time 30 min). Each exercise bout consisted of a 5-min warm-up and 5-min cool down as part of the total session. Intervals were designed to elicit a heart rate corresponding to 80–90% of VO_2_ reserve (high intensity intervals) and 50–60% VO_2_ reserve (active recovery). Heart rate was recorded continuously during each session (Polar OH1, Polar, USA). Speeds did not exceed walking pace (1–4.8 mph), and if heart rate was not achieved by the end of the interval, the gradient (1–15%) was elevated to increase heart rate. High intensity intervals were between 60 and 90 s followed by active recovery intervals of a similar duration. Rather than controlling each session for energy expenditure, we adjusted total intervals per session so that the aerobic exercise duration each session was 30 min. Ratings of perceived exertion were detailed at the end of each high intensity interval. Resistance training exercises targeted major muscle groups in the upper and lower body using the leg press, chest press, and seated row machines. Following a warm-up of 10 repetitions at 40–60% 1 RM, sets consisted of as many repetitions possible at 70% 1 RM were completed. Once participants were able to perform 20 repetitions or more, the weight was increased for the next session by 2–5 kg. We adopted a flexible scheduling protocol to ensure participants could complete all 3 prescribed sessions/week (6 am to 6 pm, any day of 5 days per week or per request of the participant), and supervision by an exercise physiologist. If a participant was unable to complete the entire session, the number of minutes completed in that session was documented.

**Table 1:** Exercise Prescription and results of the intervention.

	High-intensity interval	Low-intensity interval
**Prescribed**		
Intensity (% VO_2peak_)	80–90	50–60
Heart rate (bpm)	139 (19)–149 (21)	110 (13)–119 (14)
Aerobic exercise		
Each interval (sec)	60–90	60–90
Warm-up + cool down (min)	10	
Total time/week (min)	90	
Total sessions (N)	36	
Total exercise exposure (min)	1080	
Resistance exercise		
Total time/week (min)	60	
Total sessions (N)	24	
Total exercise exposure (min)	720	
Baseline 1 RM (kg)		
Leg press	137.9 (53.5)	
Chest press	43.2 (26.9)	
Seated row	51.2 (24.9)	
**Adherence**		
Total exercise adherence (%Rx sessions)	99 (3.6)	
Total minutes of exercise/week (mins/wk)	148.5 (5.4)	
**Actual aerobic**		
Intensity (% VO_2peak_)	89 (2.8)	72 (4.8)
Heart rate (bpm)	142 (19)	127 (6)
Exercise adherence (%Rx sessions)	99.4 (2.9)	
**Actual resistance**		
Exercise adherence (%Rx sessions)	98.3 (6.0)	
Final 1 RM (kg)		
Leg press	179.5 (53.2)	
Chest press	63.0 (39.2)	
Seated row	68.3 (31.3)	

VO_2peak_ (Aerobic Capacity in mL/kg/min); bpm (beats per minute); min (minutes); Rx (prescription); kg (kilogram); RM (repetition maximum). Data are mean (SD).

### Blood samples

Participants arrived for blood draws at the same time before and after the intervention having consumed the same diet the day prior to the visit. They were instructed to not eat or drink anything besides water the morning of their visit. A total of 50 mL blood was taken for immediate processing. Complete blood differential counts were measured in EDTA anticoagulated whole blood using a clinical hematology analyzer (Sysmex, USA).

### Immune cell isolation

Peripheral blood mononuclear cells (PBMCs) were isolated within 1 h of collection, from heparin anticoagulated blood following density gradient centrifugation, as previously described^27^. Briefly, blood was diluted 1:1 with sterile phosphate buffer saline (PBS) before being layered on Ficoll-Paque Plus and processed according to manufacturer guidelines (Sigma-Aldrich, MO, USA). PBMC viability was determined by trypan blue exclusion and was consistently > 98%. A sample of PBMCs were processed for cryopreservation using standard methods (fetal bovine serum (FBS) + 10% DMSO) and stored in liquid nitrogen.

We isolated CD19 + B-cells from 5 mL of blood using negative selection density gradient centrifugation using the manufacturer’s guidelines (RosetteSep Human B Cell Enrichment Kit, STEMCELL technologies, MA, USA). Briefly, blood was incubated for 20 min with a tetrameric antibody complex that recognizes non-B-cells and glycophorin A on RBCs. Blood was diluted with sterile PBS and layered on Ficoll-Paque. Following centrifugation, only the negatively enriched B-cells at the interphase were removed, washed and analyzed for purity using CD19 + flow cytometry staining and viability by trypan exclusion. Purity and viability were consistently > 98%. A sample of B-cells was processed for cryopreservation, with the remaining cells used for the target population in the NK-cell cytotoxicity assays (see below). These cells were maintained at 1 × 10^6^ cells/mL in complete media (RPMI 1640 + 2 mM L-glutamine, 100 U/mL penicillin, 100 µg/mL streptomycin, with 10% FBS).

### Measurement of PBMC subsets by flow cytometry

We determined frequencies of PBMC subset (T-cells, B-cells, CLL-cells, NK-cells and monocytes) using a 3-laser BD FACSCanto II (BD Bioscience, USA) flow cytometer. Flow cytometry was completed at the Duke Cancer Institute Core Facility, which maintained daily quality controls of the machine. We analyzed 10,000 cells of interest and assessed the data with FCS Express 6 (FCS Express, USA). Lymphocytes and monocytes were gated using standard forward versus side scatter properties. We identified NK-cells as CD3^neg^/CD56+, T-cells as either CD3+/CD4+ or CD3+/CD8+, B-cells as CD3^neg^/CD19+/CD5^neg^, CLL-cells as CD3^neg^/CD19+/CD5+, and monocytes as CD14+ cells. We further classified monocytes as classic (CD14+/CD16^neg^), intermediate (CD14+/CD16+) or non-classic (CD14+/CD16++). PBMCs (1 × 10^6^ cells/mL in PBS/1% BSA) were incubated on ice for 30 min with combinations of the following fluorochrome conjugated monoclonal antibodies, or relevant concentration-matched isotype controls. All antibodies were titrated prior to assessing samples and single color and flow minus one (FMOs) tubes were used for compensation. We used 0.75 µg/mL CD3 Pacific Blue (Clone UCHT1; BD Bioscience, NC, USA)**,** 0.2 µg/mL CD56-PE (Clone 5.1H11, BioLegend, CA, USA), 1 µg/mL CD4-PE (Clone OKT4; BioLegend), 10 µg/mL CD8 FITC (Clone OKT8; Thermofisher, MA, USA), 1.5 µg/mL CD19-APC-Cy7 (Clone HIB19; BioLegend), 3 µg/mL CD5-APC (Clone UCHT2; BioLegend), 1 µg/mL CD14-PcB (Clone TuK4; Thermofisher), and CD16-FITC (Clone 3G8, BD Bioscience). After incubations, we washed samples once and resuspended them in 300µL of 4% formaldehyde for 20 min, before flow cytometry.

### NK-cell cytolytic activity

We assessed NK-cell cytotoxicity (NKCC) by three-color flow cytometry using adapted methods of Hazeldine et al*.*^28^. Briefly, we isolated NK-cells from PBMC samples by negative magnetic selection using MACS® technology (Human NK Cell Isolation Kit, Miltenyi Biotec, MD, USA), and resuspended them in complete media at 1 × 10^6^ cells/mL. Because NK-cell frequency is lower in CLL patients, we used multiple purifications to attain a minimum of 1 × 10^6^ NK-cells. NK-cells were assessed for purity via CD3^neg^/CD56^+^ flow cytometry staining and viability by trypan exclusion. Purity and viability were consistently > 95%. Target cells were the MHC-I deficient cell line K562 (ATCC, VA, USA), the EBV transformed stable CLL-like cell line OSU-CLL, from the Byrd Lab at Ohio State University^29^, and autologous B-cells isolated from PBMC samples (see above). K562 and OSU-CLL cells were maintained in complete media at 37 °C in a humidified 5% CO_2_ incubator. We stained K562 and OSU-CLL cells with anti-CD71-PE-Cy7, while autologous B-cells were stained with anti-CD19-APC-Cy7 and anti-CD5-APC, prior to the assay beginning, and similar to previous methods^30^. K562 and OSU-CLL cell passage was identical for pre and post-intervention. NK-cells and target cells were co-cultured at effector: target (E:T) ratios of 10:1, 5:1, and 0:1 (spontaneous death) for 4 h at 37 °C/5% CO_2_. After incubation, we pelleted cells and resuspended them in PBS/1% BSA containing anti-CD56-PE for 20 min on ice. Cells were then washed and resuspended in PBS, and stained for 5 min with 125 nM sytox® blue dead cell stain (ThermoFisher, MA, USA) before immediate analyses by flow cytometry. NKCC was determined by the number of lysed target cells (defined as sytox blue positive) within a population of 5000 cells, with percentage-specific lysis calculated as the number of lysed target cells (TL) minus lysed target cells without effector cells (SL), divided by the number of cells recorded (C#) and multiplied by 100 ((TL-SL/C#) × 100)^28^.

### Disease characteristics

We obtained clinical indices from patients’ medical records. Indices included disease duration, cytogenetics, IGHV mutation status, and CD38 expression. The CLL-IPI score, calculated as previously described^31^. We prepared plasma from blood immediately after venesection, and samples were stored at minus 80 °C until analyzed. All plasma analyses were completed within the Biomarker Core Facilities of the Duke Molecular Physiology Institute. Concentrations of soluble CD20 (sCD20: pg/mL) and intercellular adhesion molecule 1 (ICAM-1: ng/mL) were determined in duplicate using a human sandwich immunoassay according to the manufacturer’s instructions (Meso Scale Discovery, Rockville, MD). β2-microglobulin (B2M: µg/mL) was determined in duplicate using a commercially available ELISA (R&D Systems, Minneapolis, MN). The lower limits of detection (LLOD) were sCD20 (30.5 pg/mL), ICAM-1 (2.60 ng/mL), and B2M (0.132 µg/mL). All samples had concentrations greater than the LLOD with the exception of sCD20, for which 81% of samples were above the LLOD.

### Feasibility, fidelity, compliance, and safety

We defined feasibility by participant adherence. Specifically, feasibility was achieved if > 70% of participants assigned to HIIT could complete > 75% (i.e., 45 of 60 sessions) of prescribed sessions and complete > 75% (i.e., 112.5 of 150 min/week) of prescribed weekly minutes. Before and after every session, adverse events were monitored by determining if participants had any injuries, nausea, infections, pain, and shortness of breath. We confirmed fidelity if > 80% of all prescribed testing was completed. Fidelity was calculated as the percentage of all tests each participant completed at baseline, and at the follow-up after the total number of required tests were completed (e.g. aerobic fitness, muscle strength, body composition, blood samples). We confirmed compliance if > 80% of all high-intensity intervals were completed at a heart rate corresponding to at least 80% VO_2peak_. Each high-intensity interval average heart was recorded and compared against prescribed heart rates determined from aerobic fitness testing.

### Statistics

This pilot, single-center study in which participants were not randomly assigned was quasi-experimental. All analyses were conducted using IBM SPSS version 23.0 (Armonk, NY, United States). Normality was assessed using Kolmogorov–Smirnov analysis. For variables violating normality, natural log transformation was performed or non-parametric analyses was employed. Descriptive statistics are shown for baseline characteristics and compared across groups using independent T-tests (or Wilcoxon sum of ranks) for continuous variables and goodness-of-fit chi-square for categorical. For each participant, adherence was calculated as (1) the number of sessions attended/number of prescribed sessions and multiplied by 100 and (2) the average number of weekly exercise minutes/number of prescribed weekly minutes and multiplied by 100. Differences in mean percent change by group in fitness and immune function was assessed by ANCOVA, where the baseline value of each dependent variable was included as a covariate. Because of the risk of recruitment bias, we have not reported *p*-values throughout. Instead, statistics are presented as mean ± standard deviation (SD), or mean (SD) or mean differences and 95% confidence intervals, unless otherwise indicated, and presented with effect sizes. Effect sizes were calculated as Hedges’ *G* (*g*) due to differences in group N’s for comparisons between baseline characteristics, and mean intervention differences^32,33^. We considered small, medium and large effect sizes if *g* equated to 0.2, 0.5 and 0.8, respectively.

## Results

### Participants

Baseline group demographics, disease related blood markers and disease characteristics are presented in Table 2. There were sixteen participants [8 men and 8 women, mean age of 64.9 (range: 49–79) years] with stable, confirmed treatment-naïve CLL who completed the 12-week study. Mean years since diagnosis was 6.3 years (range: 0.5–24 years). Participants were mostly Rai stage 0 or 1 (81.2%), and those with CLL-IPI scores were mostly 0 or 1. One participant in the control group had a CLL-IPI score of 3 and one in the HIIT group a score of 2. Fourteen (87.5%) participants had cytogenetics defined as either normal, del13q, monosomy 13, trisomy 12 and/or del11q. *IGHV* status was assessed in ten participants (62.5%) and *TP53* was assessed in six participants (37.5%). Groups were similar at baseline for physical fitness and most immune cell characteristics (Table 3). Large effect sizes were observed between groups for absolute monocyte counts, which were 37.5% lower in the HIIT group (95% CI [− 0.6, − 0.01], *g* = 1.08). This resulted in the ratio of T-cells to monocytes being 40.7% higher in the HIIT group (95% CI [− 0.9, 3.1], *g* = 0.82). Large effect sizes were observed between groups for the frequency (% of lymphocytes) of CD8 + T-cells, which were 6.8% lower in the HIIT group (95% CI [− 15.2, 1.6], *g* = 0.92) and the frequency of intermediate monocytes, which were 1.6% lower in the HIIT group (95% CI [− 3.7, 0.5], *g* = 0.82). Absolute counts of NK-cells were 40.1% lower in the HIIT group (95% CI [− 0.5, 0.1], *g* = 0.89), while NK-cell specific lysis of autologous B-cells was 11.4% lower in the HIIT group (95% CI [− 1.4, 21.5], *g* = 1.22).

**Table 2:** Baseline and percentage changes upon study completion for demographics and clinical characteristics of participants completing the intervention.

	Controls (N = 6)		HIIT (N = 10)		Mean difference at baseline (95% C.I.)	Effect size at baseline (*g*)	Mean difference for % change (95% C.I.)	Effect size for % change (*g*)
	Baseline	12-week % change	Baseline	12-week % change				
**Demographics**								
Age (yrs.)	66.5 (7.1)		63.9 (10.8)		− 2.6 (− 12.9, 7.7)	0.27		
Sex (M/F)	4/2		4/6					
Weight (kg)	77.6 (9.7)	− 0.3 (2.1)	79.4 (24.0)	0.6 (1.4)	1.7 (− 20.5, 24.0)	0.08	− 1.0 (− 2.9, 1.0)	0.53
BMI (kg/m^2^)	25.4 (3.1)	− 0.4 (2.1)	27.3 (6.6)	0.5 (1.4)	2.1 (− 4.2, 8.2)	0.36	− 1.1 (− 3.0, 0.8)	0.53
Body Fat (%)	31.0 (10.3)	− 7.2 (9.1)	37.7 (10.3)	− 4.9 (12.5)	6.8 (− 4.6, 18.2)	0.62	0.3 (− 16.8, 17.3)	0.20
Lean Mass (kg)	53.7 (10.5)	2.0 (2.8)	48.2 (12.4)	3.1 (5.2)	− 5.5 (− 18.5, 7.5)	0.44	− 0.7 (− 7.3, 5.9)	0.24
HbA1c (%)	5.8 (0.4)	− 0.8 (2.9)	5.6 (0.3)	1.4 (1.6)	− 0.3 (− 0.6, 0.1)	0.72	**− 1.7 (− 4.3, 0.8)**	**1.02**
**Disease related blood markers**								
WBC (× 10^3^/µL)	39.5 (28.9)	3.7 (14.5)	23.9 (18.8)	4.1 (11.4)	− 15.5 (− 40.9, 9.9)	0.64	1.2 (− 14.0, 16.3)	0.03
Lymphocytes	35.1 (28.8)	− 3.0 (15.8)	19.8 (18.9)	1.0 (18.0)	− 15.3 (− 40.7, 10.2)	0.63	− 2.5 (− 23.6, 18.5)	0.23
Monocytes	0.8 (0.3)	89.8 (191.9)	0.5 (0.3)	86.4 (240.7)	**− 0.3 (− 0.6, − 0.0)**	**1.08**	− 54.1 (− 348.0, 240.0)	0.02
Neutrophils	3.4 (1.1)	59.8 (121.4)	3.4 (1.2)	17.4 (52.5)	− 0.0 (− 1.4, 1.3)	0.03	43.8 (− 41.4, 129.0)	0.51
Neutrophil: T-cell	1.8 (0.8)	226.4 (491.5)	1.9 (0.9)	49.7 (78.1)	0.2 (− 0.9, 1.0)	0.22	137.3 (− 178.0, 453.0)	0.59
T-cell: Monocyte	2.7 (1.0)	− 20.2 (64.8)	3.8 (2.2)	− 32.8 (27.5)	**1.5 (− 0.9, 3.1)**	**0.82**	15.4 (− 44.4, 75.1)	0.28
Platelets (× 10^3^/µL)	212.0 (35.0)	2.7 (7.9)	193.0 (63.3)	− 0.4 (5.1)	− 19.0 (− 80.7, 42.7)	0.33	3.5 (− 4.1, 11.1)	0.50
Hemoglobin (g/dL)	14.2 (1.4)	0.7 (4.5)	13.6 (1.5)	0.7 (3.2)	− 0.6 (− 2.2, 0.9)	0.42	0.6 (− 3.4, 4.7)	0.00
RBC (× 10^6^/µL)	4.6 (0.4)	0.7 (3.3)	4.5 (0.4)	− 1.8 (9.4)	− 0.1 (− 0.5, 0.3)	0.29	1.6 (− 7.0, 10.2)	0.32
Hematocrit (%)	42.5 (3.4)	1.3 (3.7)	41.0 (3.6)	0.8 (3.8)	− 1.5 (− 5.4, 2.4)	0.39	1.0 (− 3.3, 5.3)	0.13
β2-microglobulin (µg/mL)	2.1 (0.8)	− 0.1 (12.6)	2.5 (1.6)	5.6 (12.7)	0.4 (− 1.2, 1.9)	0.26	− 9.0 (− 20.0, 2.0)	0.45
ICAM-1 (ng/mL)	532.4 (128.7)	4.3 (8.9)	564.2 (216.8)	10.8 (16.8)	9.3 (− 175.8, 239.5)	0.05	− 7.1 (− 15.0, 0.8)	0.45
sCD20 (pg/mL)	202.2 (168.9)	105.5 (186.8)	133.0 (124.6)	92.9 (143.1)	− 87.1 (− 226.5, 88.1)	0.59	59.7 (− 119.6, 239.1)	0.08
**Disease characteristics**								
Disease duration (yrs.)	4.6 (4.3)		7.3 (8.7)		2.7 (− 5.6, 10.9)	0.34		
Rai Stage (N (%))^#^						0.10		
0	4 (66.7%)		7 (70%)					
I	1 (16.7%)		1 (10%)					
Unknown	1 (16.7%)		2 (20%)					
CLL-IPI Score (N (%))^#^						0.42		
0–1	2 (33.3%)		2 (20%)					
2–3	1 (16.7%)		1 (10%)					
Unknown	3 (50%)		7 (70%)					
Cytogenetics (N (%))^#^						0.66		
Normal	1 (16.7%)		4 (40%)					
del13q	4 (66.7%)		2 (20%)					
Tri 12 and /or del11q	0		3 (20%)					
Monosomy 13	1 (16.7%)		0					
Unknown	0		2 (20%)					
*TP53* Unmutated (N (%))^#^	1 (16.7%)		5 (50%)			0.33		
Unknown	5 (83.3%)		5 (50%)					
*IGHV* Mutated (N (%))^#^	3 (50%)		3 (30%)			0.21		
Unknown	2 (33.3%)		4 (40%)					
CD38 > 30% (N (%))^#^	1 (16.7%)		0			0.38		
Unknown	2 (33.3%)		6 (60%)					

HbA1c (hemoglobin A1c); RBC (red blood cells); ICAM-1 (intercellular adhesion molecule-1); IGHV (immunoglobulin heavy chain variable region). ^#^Chi-square test with Cramer’s V effect size. Data are mean (SD) or mean difference (95% C.I.), unless otherwise indicated. Bolded effect sizes indicate large effect sizes for mean differences between groups.

**Table 3:** Baseline fitness and immune characteristics of participants completing the intervention.

	Controls	HIIT	Mean Diff (95% C.I.)	Effect size (*g*)
**Fitness characteristics**				
Aerobic capacity				
VO_2peak_ (mL/kg/min)	26.7 (6.1)	28.5 (5.5)	1.8 (− 4.8, 8.4)	0.30
VO_2peak_ (L/min)	2.1 (0.4)	2.2 (0.7)	0.1 (− 0.6, 0.8)	0.14
VO_2peak_ (mL/kg lean mass/min)	38.5 (5.7)	44.2 (7.8)	5.6 (− 2.4, 13.7)	0.75
Time-to-exhaustion (sec)	706.3 (175.8)	641.3 (241.2)	− 65.0 (− 308, 179)	0.28
Estimated 1RM (kg)				
Leg press	133.5 (30.0)	137.9 (53.5)	4.4 (− 47.1, 55.9)	0.09
Chest press	48.8 (20.2)	43.2 (26.9)	− 5.6 (− 35.8, 24.7)	0.21
Seated row	65.7 (27.8)	51.2 (24.9)	− 14.5 (− 43.2, 14.3)	0.53
Muscular endurance (repetitions at 70% 1RM)				
Leg press	20.6 (4.9)	20.3 (5.7)	− 0.3 (− 6.8, 6.1)	0.05
Chest press	14.8 (1.7)	14.4 (3.5)	− 0.4 (− 4.4, 3.7)	0.10
Seated row	15.8 (1.3)	15.7 (3.8)	− 0.1 (− 4.0, 3.8)	0.03
Steps/Day	10,008 (2,197)	8,281 (4,262)	− 1626 (− 6063, 2810)	0.41
**Immune characteristics**				
B-cells				
CD19+ (× 10^3^/µL)	26.3 (18.1)	15.3 (16.4)	− 11.0 (− 30.5, 8.4)	0.61
CD19+ (% Lymphocytes)	78.9 (13.4)	73.8 (12.4)	− 5.2 (− 19.8, 9.4)	0.38
CD19+/CD5+ (% Lymphocytes)	78.1 (14.3)	72.4 (12.4)	− 5.7 (− 20.7, 9.3)	0.41
T-lymphocytes				
CD3+ (× 10^3^/µL)	2.1 (0.8)	1.7 (0.4)	− 0.4 (− 1.1, 0.3)	0.58
CD3+ (% Lymphocytes)	10.4 (7.3)	15.5 (9.9)	5.2 (− 5.1, 15.4)	0.54
CD3+/CD56+ (% Lymphocytes)	0.8 (0.4)	1.1 (1.0)	0.3 (− 0.7, 1.3)	0.35
CD3+/CD4+ (% CD3+)	64.9 (10.5)	66.7 (6.4)	1.8 (− 8.0, 11.5)	0.21
CD3+/CD8+ (% CD3+)	33.2 (6.3)	26.4 (7.2)	**− 6.8 (− 15.2, 1.6)**	**0.92**
**Monocyte subtypes (%)**				
CD14+/CD16^neg^ (Classic)	82.9 (7.2)	85.0 (6.8)	2.2 (− 5.7, 10.1)	0.30
CD14+/CD16+ (Intermediate)	5.9 (2.3)	4.3 (1.5)	**− 1.6 (− 3.7, 0.5)**	**0.82**
CD14+/CD16++ (Non-Classic)	7.5 (4.4)	7.1 (6.5)	− 0.3 (− 4.1, 0.9)	0.06
NK-cells				
CD3^neg^/CD56 + (× 10^3^/µL)	0.6 (0.3)	0.3 (0.2)	**− 0.2 (− 0.5, 0.1)**	**0.89**
CD3^neg^/CD56^dim^ (%Lymph.)	2.7 (2.5)	2.7 (2.5)	0.0 (− 2.9, 2.9)	0.01
CD3^neg^/CD56^bright^ (%Lymph.)	0.1 (0.1)	0.1 (0.1)	0.0 (− 0.1, 0.1)	0.25
Perforin (MFI)	981 (194)	810 (315)	− 171 (− 483, 142)	0.59
Granzyme B (MFI)	567 (413)	396 (310)	− 172 (− 574, 230)	0.46
NK-cell cytotoxicity (specific lysis (%))				
K562 cell line	34.6 (10.0)	33.0 (20.8)	− 1.6 (− 21.5, 18.3)	0.09
OSU-CLL cell line	7.6 (4.0)	11.6 (8.9)	4.1 (− 4.4, 12.5)	0.51
Autologous CD19 + B-cell	− 19.1 (7.0)	− 7.6 (9.7)	**11.4 (1.4, 21.5)**	**1.22**

Data are mean (SD) or mean difference (95% C.I.), unless otherwise indicated. Bolded effect sizes indicate large effect sizes for mean differences between groups.

Recruitment rate was 76% over the 18-month period (Fig. 1). A total of six screened participants declined participation because they did not have enough time for participation (n = 4) or did not want additional blood taken (n = 2). Nineteen participants were consented into the study. One participant had an abnormal CPET and was referred to cardiology before being excluded from the study. Of the 18 participants assigned to the study groups (11 to HIIT, 7 to Control), 16 completed the study. One Control participant developed a chronic skin infection (hives) shortly after the CPET, but before training begun, and was excluded. One participant in the HIIT group failed to come to training or to respond to repeated attempts to be contacted and was excluded. The remaining 16 participants completed the 12-week program.

### Effects of HIIT

Upon completing the 12-weeks, we observed similar percent changes between Controls and HIIT for disease related blood markers and most demographics, with HIIT having a large effect on HbA1c that was 1.7% higher than Controls (Table 2: 95% CI [− 4.3, 0.8], *g* = 1.02).

#### Physical function responses to HIIT (Fig. 2)

##### Aerobic capacity

**Figure 2:**
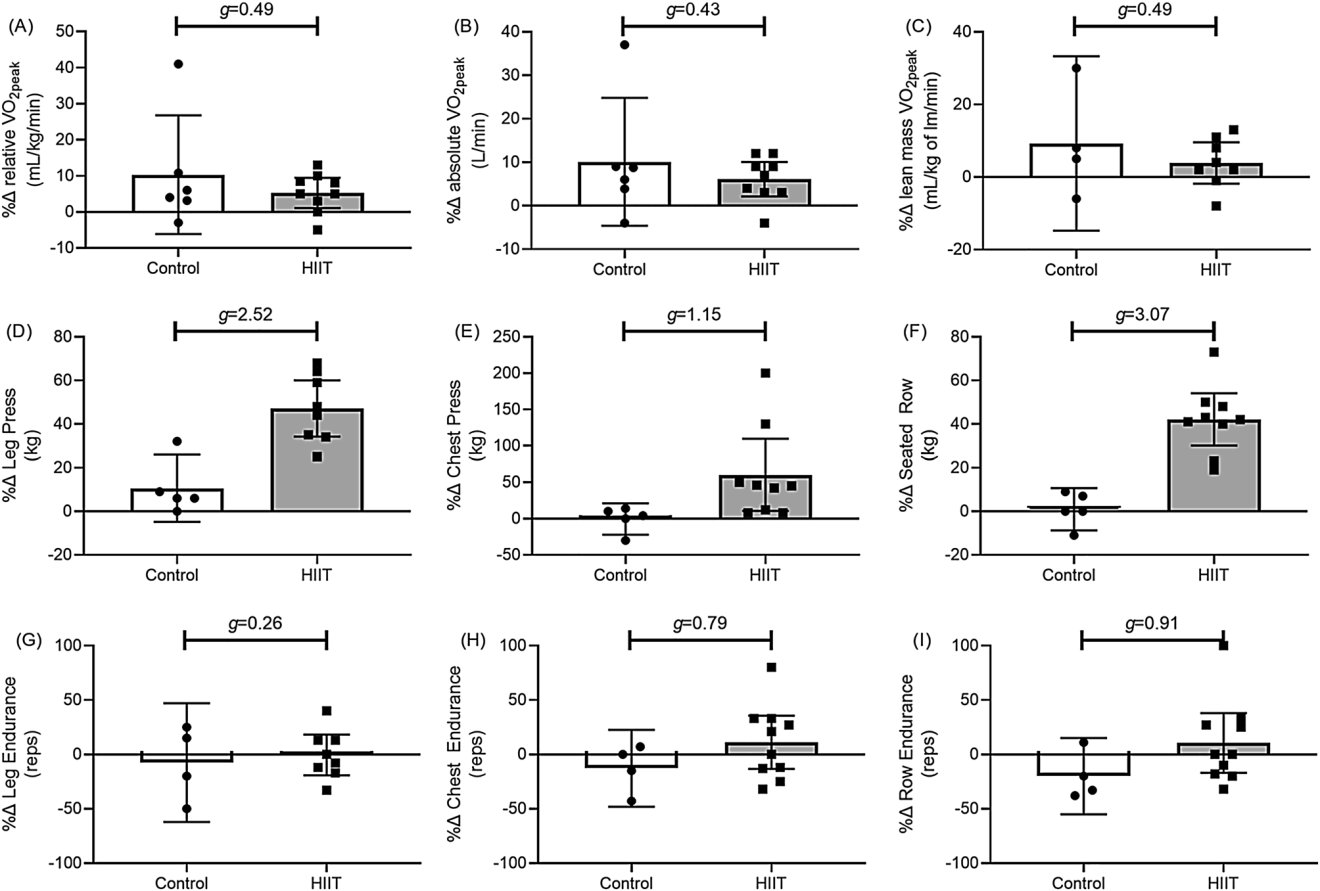
Mean (95% C.I.) percentage change (%∆) with Hedges G (*g*) group differences between HIIT and controls for cardiorespiratory fitness (**A**–**C**), estimated 1 repetition maximum strength (**D**–**F**), and number of repetitions completed at 70% of 1 RM (**G**–**I**).

HIIT had a small-medium effect on aerobic capacity. The percentage change for relative aerobic capacity was 3.8% lower than Controls following HIIT (Fig. 2A: 95% CI [− 15.8, 8.1], *g* = 0.49), while absolute aerobic capacity was 3.5% lower following HIIT (Fig. 2B: 95% CI [− 14.3, 7.3], *g* = 0.43), and lean mass adjusted relative aerobic capacity was 4.0% lower following HIIT (Fig. 2C: 95% CI [− 20.1, 12.1], *g* = 0.49).

##### Maximum strength

HIIT had a large effect on muscle strength. As compared to Controls, the percentage change for maximal leg strength was 35.4% higher following HIIT (Fig. 2D: 95% CI [17.3, 53.5], *g* = 2.52), while maximal chest strength was 56.1% higher following HIIT (Fig. 2E: 95% CI [− 8.1, 120.3], *g* = 1.15), and maximal seated row strength was 39.5% higher following HIIT (Fig. 2F: 95% CI [22.6, 56.5], *g* = 3.07).

##### Muscular endurance

HIIT had a medium-large effect on upper body muscular endurance. As participants in the HIIT group increased muscular strength (Table 1), the relative weight used to test muscular endurance increased also. As compared to Controls, the percentage change for leg muscular endurance was 10.4% higher following HIIT (Fig. 2G: 95% CI [− 27.3, 48.2], *g* = 0.26), while chest muscular endurance was 21.7% higher following HIIT (Fig. 2H: 95% CI [− 11.0, 54.3], *g* = 0.79), and seated row muscular endurance was 29.2% higher following HIIT (Fig. 2I: 95% CI [− 13.9, 72.3], *g* = 0.91).

#### NK-cell responses to HIIT (Fig. 3)

##### NK-cell numbers and frequencies

**Figure 3:**
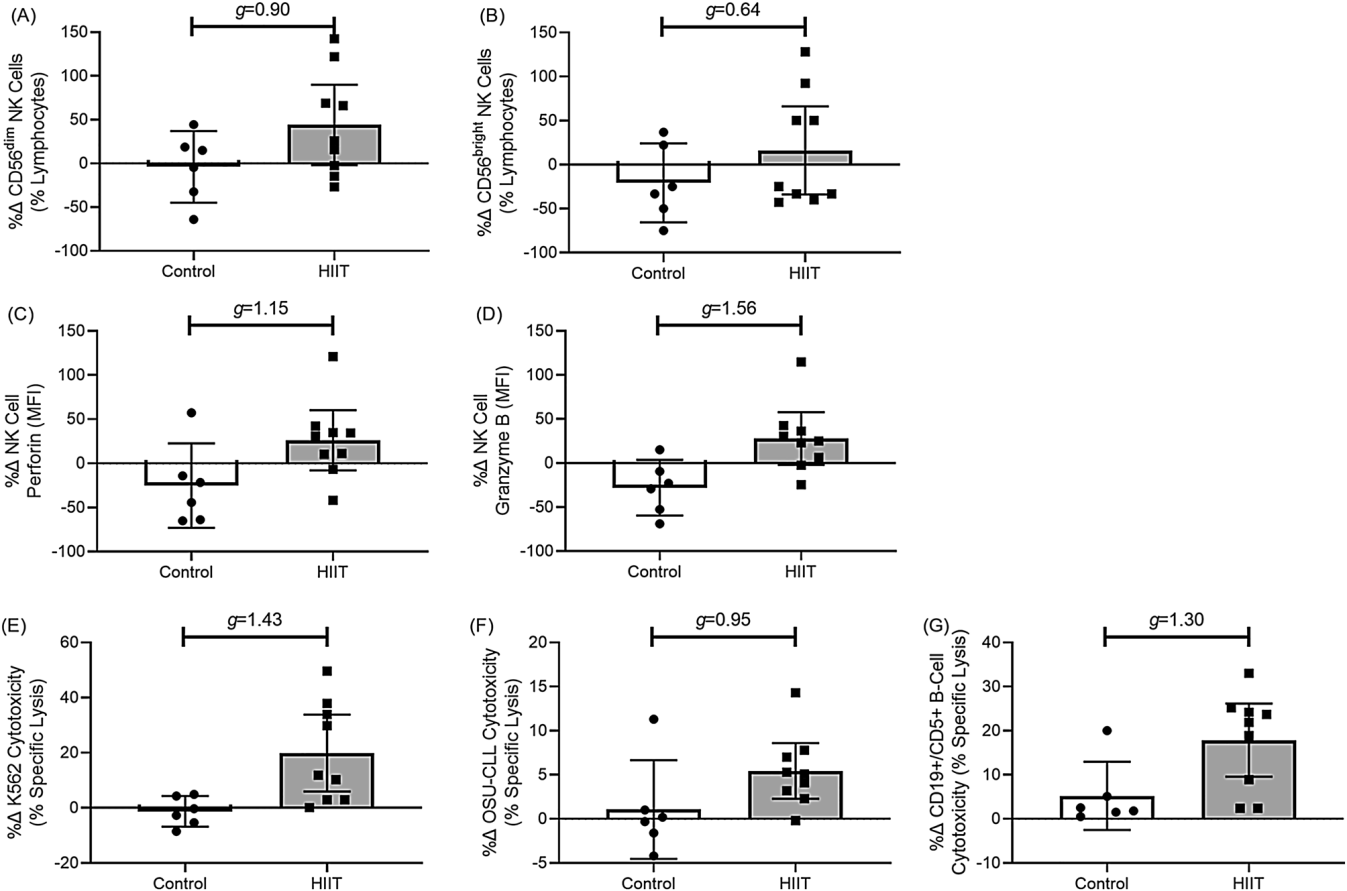
Mean (95% C.I.) percentage change (%∆) with Hedges G (*g*) group differences between HIIT and controls for CD56^dim^ NK-cell (**A**) and CD56^bright^ NK-cell (**B**) frequencies, expression (MFI) of NK-cell specific perforin (**C**) and granzyme B (**D**), and NK-cell cytotoxicity towards K562 (**E**), OSU-CLL (**F**), and autologous CD5 + B-cells (**G**).

HIIT had a large effect on NK-cell numbers and CD56^dim^ NK-cells. As compared to Controls, the percentage change for the absolute number of CD56 + NK-cells was 51.0% higher following HIIT (Data not shown: 95% CI [− 45.9, 111.0], *g* = 0.81), while the frequency of CD56^dim^ NK-cells was 47.9% higher following HIIT (Fig. 3A: 95% CI [− 14.4, 110.0], *g* = 0.90), and CD56^bright^ was 36.9% higher following HIIT (Fig. 3B: 95% CI [− 13.9, 61.8], *g* = 0.64).

##### NK-cell functions

HIIT had a large effect on NK-cell functions. As compared to Controls, the percentage change for the expression (MFI) of NK-cell specific perforin was 52.6% higher following HIIT (Fig. 3C: 95% CI [− 3.5, 108.8], *g* = 1.15), while expression of granzyme B was 53.8% higher following HIIT (Fig. 3D: 95% CI [10.1, 97.4], *g* = 1.56). As compared to Controls, the percentage change for the NK-cell specific lysis of the K562 cell line was 20.3% higher following HIIT (Fig. 3E: 95% CI [7.3, 33.3], *g* = 1.43), while NK-cell specific lysis of the OSU-CLL cell line was 3.0% higher following HIIT (Fig. 3F: 95% CI [− 1.8, 7.9], *g* = 0.95), and NK-cell specific lysis of autologous B-cells was 14.6% higher following HIIT (Fig. 3G: 95% CI 0.9, 28.4], *g* = 1.30).

#### Non-NK-cell mononuclear responses to HIIT (Fig. 4)

##### B-cell, T-lymphocyte and monocyte changes

**Figure 4:**
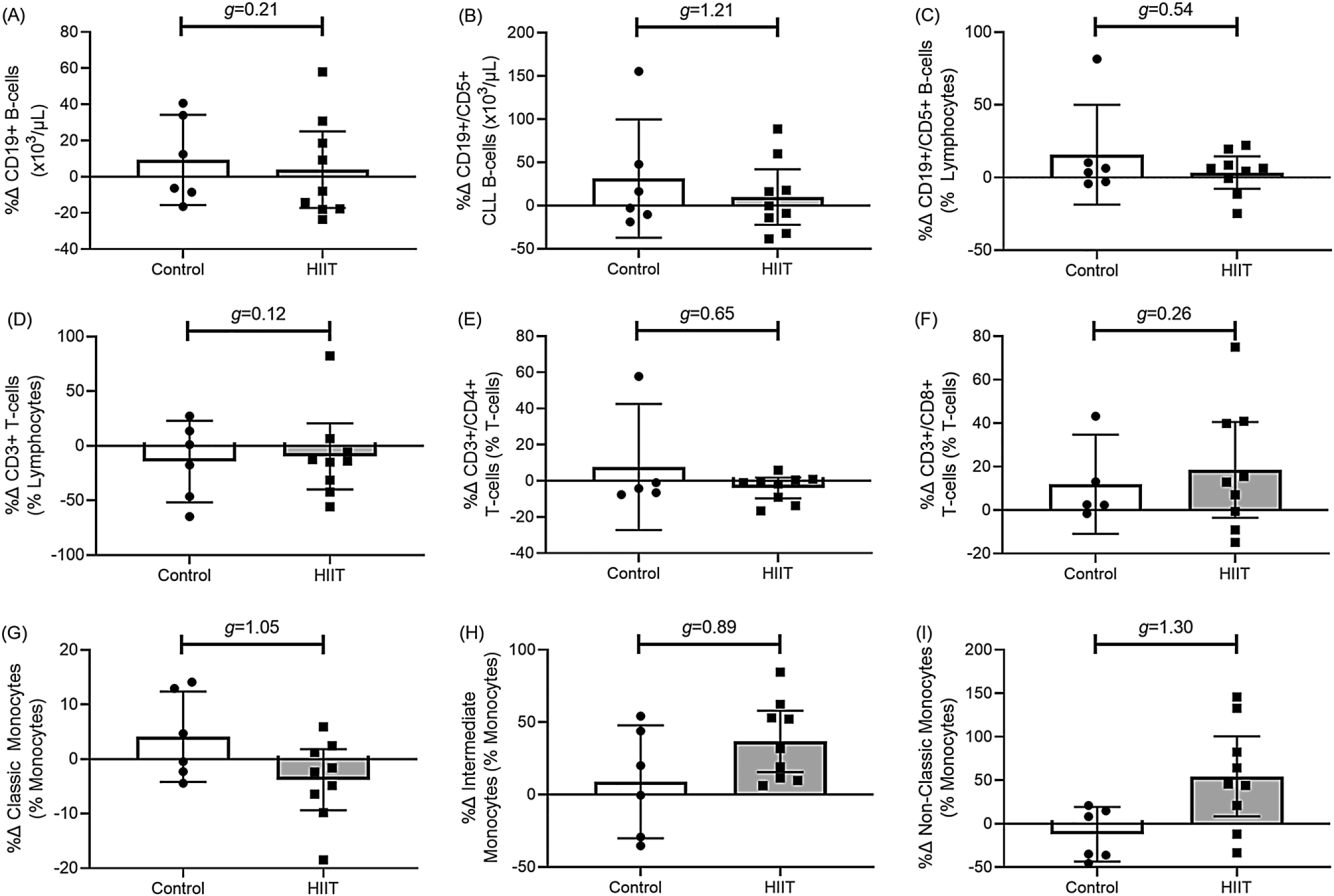
Mean (95% C.I.) percentage change (%∆) with Hedges G (*g*) group differences between HIIT and controls for B-cells (**A**–**C**), T-cells (**D**–**F**), and monocytes (**G**–**H**).

HIIT had a small-medium effect on B-cells and T-cells, and a large effect on monocytes. As compared to Controls, the percentage change for the absolute number of CD19 + B-cells was 5.7% lower following HIIT (Fig. 4A: 95% CI [− 38.8, 27.2], *g* = 0.21), while the absolute number of CD19+/CD5 + CLL-cells was 21.4% lower following HIIT (Fig. 4B: 95% CI [− 80.7, − 37.9], *g* = 1.21), and the frequency of CD19 + /CD5 + CLL-cells was 19.2% lower following HIIT (Fig. 4C: 95% CI [− 40.0, 1.6], *g* = 0.54). As compared to Controls, the percentage change for the frequency of CD3 + lymphocytes was 4.4% higher following HIIT (Fig. 4D: 95% CI [− 43.0, 51.8], *g* = 0.12), the frequency of CD3+/CD4 + lymphocytes was 10.4% lower following HIIT (Fig. 4E: 95% CI [− 31.7, 10.8], *g* = 0.65), the frequency of CD3+/CD8 + lymphocytes was 2.8% higher following HIIT (Fig. 4F: 95% CI [− 36.7, 32.6], *g* = 0.26), and the frequency of CD3+/CD56 + lymphocytes was 33.3% higher following HIIT (data not shown: 95% CI [− 37.8, 104.4], *g* = 0.66). As compared to Controls, the percentage change for the frequency of CD14+/CD16^neg^ classic monocytes was 7.6% lower following HIIT (Fig. 4G: 95% CI [− 16.7, 1.4], *g* = 1.05), while the frequency of CD14+/CD16 + intermediate monocytes was 11.4% higher following HIIT (Fig. 4H: 95% CI [− 21.7, 44.6], *g* = 0.89), and the frequency of CD14+/CD16 + + non-classic monocytes was 65.5% higher following HIIT (Fig. 4I: 95% CI [9.2, 121.7], *g* = 1.30).

### Feasibility, fidelity, compliance, and safety

Ten HIIT participants completed the study and all fulfilled feasibility criteria, with 5.0 ± 0.2 exercise sessions/week completed (99 ± 3.6%). This consisted of 3.0 ± 0.2 treadmill sessions completed and 2.0 ± 0.1 resistance sessions completed. Participants completed 148.5 ± 5.4 exercise minutes/week or 99 ± 3.6% of the prescription, with 100% of the participants completing greater than 75% of prescribed minutes. Our study fulfilled participant fidelity, with 95.0 ± 7.1% and 86.3 ± 7.1% of all required tests completed at baseline and at 12-weeks, respectively.

We determined program safety by recording the incidence and severity of pain or injuries throughout the program. There were no adverse events recorded during any of the exercise sessions. At the beginning of the study, 100% of HIIT participants reported minor muscle soreness due to the resistance and aerobic exercise but were considered normal reactions to exercise training. Incidences that required brief rescheduling of training sessions included edema in the arm (N = 1), knee pain (N = 1), upper respiratory infection (N = 1), groin tenderness (N = 1), and mild foot pain (N = 1). These participants reduced training load until the situation resolved (all < 1 week). One participant was dizzy and nauseous > 3 h following the baseline CPET and was admitted to the emergency department (ED). Following consultation with ED and oncology, it was deemed that the CPET was not the cause of the symptoms, and the participant was allowed to continue in the study after ED discharge.

Compliance was achieved, with 100% of participants completing > 80% of high-intensity intervals at the prescribed heart rate. Mean high-intensity heart rate was 142 ± 19 bpm compared to the prescription of 139 ± 19 bpm at 80% VO_2peak_ and 149 ± 21 bpm at 90% VO_2peak_.

### Ethics statement

The study involving human participants was reviewed and approved by the Duke University Medical Center Institutional Review Board. The patients/participants provided their written informed consent prior to participating in this study. Trial registration, clinicaltrials.gov, NCT04950452, 06/07/2021.

## Discussion

For the first time that we are aware of, we show that 12-weeks of supervised HIIT combined with resistance training is feasible in older adults with treatment naïve CLL. Importantly, the high adherence and compliance to HIIT were associated with large effect sizes for differences between Controls and HIIT for changes in muscle strength and innate immune cell functions. Specifically, exercise training provided larger changes in maximal muscle strength of leg and upper body muscle groups. Muscle changes were accompanied by larger changes for monocyte phenotype and NK-cell characteristics, including absolute numbers, tumor cell cytotoxicity and expression of perforin and granzyme B. Together, these data suggest that HIIT-based exercise may be an important lifestyle intervention capable of improving physical and immunological functions critical to maintaining the health of older adults with treatment naïve CLL.

Patients with cancer typically have low overall cardiorespiratory fitness and muscle strength that is associated with negative outcomes during and after therapy^34^. As such, recommendations from leading Exercise Oncologists suggest that all cancer survivors should avoid inactivity and be encouraged to safely engage in exercise training^35^. Cancer therapies affect many physiological processes of fitness and frequently prevent maintenance of adequate daily physical activity and exercise levels^34^. Although exercise interventions may counteract some therapy side effects^12–14^, treatment naïve CLL patients have never experienced cancer therapies. As such, treatment naïve CLL offers a unique opportunity to explore the effects of exercise training without the complications of therapy associated side-effects. Here, we show that 12-weeks of exercise training had a small-medium effect on cardiorespiratory fitness, albeit the change in cardiorespiratory fitness was lower for the exercise group than the control group, and a large effect on increasing muscle functions. Our muscle function results are consistent with those from Furzer and colleagues^36^ who observed significant strength improvements following 12-weeks of supervised aerobic plus resistance exercise training in hematologic cancer patients recently completing treatment. Additionally, our results may be similar to those of Courneya and colleagues who observed significant increases in lean mass following 12-weeks of supervised aerobic training in hematologic cancer patients that included CLL patients (N = 14 of 122) and some patients who did not receive treatment^14^. Although Courneya and colleagues did not assess maximal strength, the ~ 1.6% lean mass difference between the control and exercise group is similar to our study. Given that cancer treatments and advanced age are catabolic for lean tissue we now add to the literature the beneficial effects of exercise training on muscle function in cancer patients free from the deleterious effects of chemotherapy. Future studies should aim to determine whether increasing and maintaining muscle function confers protection from negative CLL outcomes including frailty, time-to-treatment and premature mortality.

Although we were not powered to detect changes in cardiorespiratory fitness, unlike Furzer et al.^36^ and Courneya et al.^14^ we did not observe group differences for cardiorespiratory fitness. Compared to our previous clinical HIIT studies^17,18,21^, our current aerobic exercise component had a similar design, with 2 additional weeks of training. Those studies had an average increase of 12.4%, while our current HIIT study increased cardiorespiratory fitness by ~ 5.3%. However, our cardiorespiratory fitness findings are similar to Persoon and colleagues who used 18-weeks of HIIT for lymphoma and myeloma patients following autologous stem cell transplant^37^. In the Persoon study, 18-weeks of HIIT improved cardiorespiratory fitness by 16–25% while the control group increased by 12–19%, with no significant differences observed between groups. The larger HIIT changes observed by Persoon are likely because of the additional 6 weeks exercise training, the 20% lower baseline cardiorespiratory fitness, and the different clinical characteristics. Additionally, similar to Persoon, the 10.3% increase we observed for our control group suggests contamination. Control group contamination is becoming more common in exercise oncology trials, especially with the recent publication of the Cancer Physical Activity Guidelines^35^. That said, in women undergoing breast cancer therapy^38^ or following therapy^39^, HIIT did not increase cardiorespiratory fitness. As such, CLL may have a yet unknown effect on the ability to promote large cardiorespiratory fitness changes and future studies should aim to determine the role of CLL on metabolic energy utilization central to cardiorespiratory fitness adaptation^40^.

Given that the majority of negative outcomes for adults with CLL are mediated by the immune system we were also interested in whether exercise training could improve components of the immune system. Although previous exercise interventions in hematologic malignancies show positive effects for aerobic fitness and strength, there is a paucity of data for immune function changes^12–14^. Important immune functions for CLL patients include anti-microbial functions and NK-cell tumor recognition and cytotoxicity^8,41^. Here, we demonstrate that HIIT had a large effect on NK-cells, including increased absolute counts, cytotoxic functions, as well as increased perforin and granzyme B expression. Since CLL patients have reduced NK-cell mediated tumor cytotoxicity, reduced expression of activatory receptors, and increased inhibitory receptor expression^41,42^, exercise training could be an effective means of reducing primary and/or secondary tumor development. In healthy adults, NK-cells are highly responsive to an acute single bout of exercise that could translate to improvements following chronic exercise training interventions^11^. During and after individual acute bouts of aerobic exercise, NK-cell functions are increased, before returning to pre-exercise levels shortly after exercise cessation^30,43–46^. For exercise training interventions, effects are less clear. Although some suggest no changes following exercise training^47^, others suggest increased NK-cell tumor killing^48^ in healthy adults. Similarly, when comparing physically active against physically inactive healthy adults, NK-cell functions can be either higher^49^ or similar^50^. Together, these data suggest that in treatment naïve CLL, exercise training improves NK-cell functions in a similar mechanism as in healthy adults. Future studies should aim to determine whether the improved NK-cell functions are maintained with regular exercise and if this translates to a longer time-to-treatment. In addition to NK-cells, we observed that HIIT had a large effect on non-classic (CD14+/CD16++) and classic (CD14+/CD16^neg^) monocytes. Specifically, non-classic monocytes were higher, while classic monocytes were lower following HIIT. These results are the opposite of previous HIIT studies noted in non-cancer participants^18,21^. The non-classic populations of monocytes are similar to M1 macrophages, and classic monocytes are the primary source of monocyte-derived dendritic cells^51^. In CLL, M1 macrophages are suggested to improve tumor outcomes due to their pro-inflammatory actions in the tumor microenvironment and reducing the balance between M2 macrophages that suppress local immune functions^52^. Future studies should aim to determine the role exercise plays on CLL patient’s monocyte functions, including terminal differentiation, phagocytosis, antigen presentation and cytokine production.

Strengths of our study include a focus on treatment naïve CLL patients with a wide range of disease duration (i.e., 6 months to 24 years). As physical dysfunction and poor physical fitness are associated with poor outcomes following commencement of treatment^10^, this group of patients reflect a pre-conditioning phase to treatment. As such, improving physical fitness and function may provide better outcomes following treatment. Additionally, our exploratory analyses of immune functions offers a unique insight into how exercise might provide improved resilience to the most common negative outcomes associated with CLL. Limitations of our study include a small number of participants and possible recruitment bias due to non-random assignment of participants to groups. Further limitations include the age of the participants, which may not reflect the more commonly older age of adults diagnosed with CLL. Our eligibility criteria reduced the generalizability of the study due to a relatively lower comorbidity burden than typically observed in adults with CLL. Specifically, we excluded individuals with underlying cardiovascular abnormalities and caution is warranted when interpreting our results or prescribing this type of exercise protocol. Future studies are required to address the generalizability of exercise training in CLL and determine its effects on all those who suffer from CLL. Critically, future randomized trials are warranted to examine the beneficial effects of HIIT on physical fitness and immune function as they relate to long-term outcomes in CLL. Specifically, future incidence of infections, secondary malignancies, and time-to-treatment.

## Conclusion

In conclusion, we demonstrate that 12-weeks of HIIT combined with muscle endurance-based resistance training for treatment naïve CLL patients is feasible, and is associated with large effects on muscle strength and normal immune cell functions. There is an increasing body of literature that links exercise training, muscle function and immune functions in older adults^11^. Our findings suggest that this may also be the case for older adults with treatment naïve chronic lymphocytic leukemia. However, as this was a small pilot study, our findings require confirmation in an adequately powered randomized trial to determine the benefits of HIIT on important physical and immunological outcomes in CLL.

## Data Availability

The datasets generated during the present study are not publicly available, owing to the risk of disclosure or deduction of private individual information, but they are available from the corresponding author on reasonable request.

## Abbreviations

B2M: β2-microglobulin
CD: Cluster of differentiation
CLL: Chronic lymphocytic leukemia
CLL-IPI: Chronic lymphocytic leukemia-International Prognostic Index
CPET: Cardiopulmonary exercise test
DMSO: Dimethyl sulfoxide
DPBS: Dulbecco’s phosphate buffered saline
EBV: Epstein Barr virus
EDTA: Ethylenediaminetetraacetic acid
ER: Emergency room
FBS: Fetal bovine serum
FMO: Flow minus one
HIIT: High intensity interval training
ICAM-1: Intercellular adhesion molecule 1
IGHV: Immunoglobulin heavy chain gene
IL: Interleukin
NK-cell: Natural killer cell
PBS: Phosphate buffered saline
OSU-CLL: Ohio State University-chronic lymphocytic leukemia
PBMC: Peripheral blood mononuclear cell
RPMI: Roswell Park Memorial Institute Medium
VO2peak: Estimated peak volume of oxygen consumption
